# Supplementary material for: Employment among Childhood Cancer Survivors: A Systematic Review and Meta-Analysis
Source: Cancers (Basel). 2022 Sep 22;14(19):4586. doi: 10.3390/cancers14194586 (PMC9559689; doi:10.3390/cancers14194586)
Supplement: Supplementary file 1 [file cancers-14-04586-s001.zip › cancers-1850829-supplementary.pdf]

cancers

MDPI

File S1. Search strategy on the Medline PubMed Database.

("Survivors"[Mesh] OR "Cancer Survivors"[Mesh] OR surviv*[Tiab] OR surviv*[Text Word])

AND ("Pediatrics"[Mesh] OR "Child"[Mesh] OR "Child, Preschool"[Mesh] OR pediatric*[Tiab] OR pediatric*[Text Word] OR child*[Tiab] OR child*[Text Word] OR "preschool child*"[Tiab] OR "preschool child*"[Text Word] OR "school child*"[Tiab] OR "school child*"[Text Word] OR childhood[Tiab] OR childhood[Text Word] OR "Adolescent"[Mesh] OR adolescent*[Tiab] OR adolescent*[Text Word] OR "Young Adult"[Mesh] OR "young adult*"[Tiab])

AND ("Neoplasms"[Mesh] OR neoplasm*[Tiab] OR cancer*[Tiab] OR tumor*[Tiab] OR tumour*[Tiab] OR neoplasm*[Text Word] OR cancer*[Text Word] OR tumor*[Text Word] OR tumour*[Text Word] OR "Carcinoma"[Mesh] OR "Sarcoma"[Mesh] OR carcinoma[Tiab] OR sarcoma[Tiab] OR carcinoma[Text Word] OR sarcoma[Text Word] OR "Leukemia"[Mesh] OR leukemia[Tiab] OR leukemia[Text Word] OR "Lymphoma"[Mesh] OR lymphoma[Tiab] OR lymphoma[Text Word] OR "Hematologic Neoplasms"[Mesh] OR "hematologic neoplasm*"[Tiab] OR "hematologic cancer*"[Tiab] OR "hematologic tumor*"[Tiab] OR oncolog*[Tiab])

AND ("Employment"[Mesh] OR employment[Tiab] OR employment[Text Word] OR "Unemployment"[Mesh] OR unemployment[Tiab] OR unemployment[Text Word] OR "Occupational Health Services"[Mesh] OR "Occupational Health Service*"[Tiab] OR "work ability"[Text Word] OR "Absenteeism"[Mesh] OR absenteeism[Tiab] OR absenteeism[Text Word] OR "employment rate*"[Tiab] OR "employment rate*"[Text Word] OR "Presenteeism"[Mesh] OR presenteeism[Tiab] OR presenteeism[Text Word] OR "work productivity"[Tiab] OR "work productivity"[Text Word] OR "Work"[Mesh] OR "Work Schedule Tolerance"[Mesh] OR "work schedule"[Tiab] OR "work schedule"[Text Word] OR "Work Capacity Evaluation"[Mesh] OR "work capacity"[Tiab] OR "work capacity"[Text Word] OR "Work Engagement"[Mesh] OR "work engagement"[Tiab] OR "work engagement"[Text Word] OR "Work-Life Balance"[Mesh] OR work-life[Tiab] OR work-life[Text Word] OR "Work Performance"[Mesh] OR "work performance"[Tiab] OR "work performance"[Text Word] OR "Employee Performance Appraisal"[Mesh] OR "Workplace"[Mesh] OR "Workload"[Mesh] OR workplace[Tiab] OR workplace[Text Word] OR workload[Tiab] OR workload[Text Word] OR "Job Satisfaction"[Mesh] OR "Workflow"[Mesh] OR workflow[Tiab] OR workflow[Text Word] OR "job satisfaction"[Tiab] OR "job satisfaction"[Text Word] OR "Occupational Groups"[Mesh] OR employee*[Tiab] OR employee*[Text Word] OR worker*[Tiab] OR worker*[Text Word] OR personnel[Tiab] OR personnel[Text Word] OR "fit for work"[Tiab] OR "Sick Leave"[Mesh] OR "sick leave"[Tiab] OR "work disability"[Tiab] OR "sickness absence"[Tiab])

Table S1. Quality Assessment of included studies [13–101].

Author, Year	Q1	Q2	Q3	Q4	Q5	Q6	Q7	Q8	Q9	Total
Ahomaki et al. 2016	1	1	1	1	0	1	0	1	0	6
Armstrong et al. 2013	1	1	1	1	1	1	1	0	0	7
Berbis et al. 2016	1	1	1	1	1	1	0	0	1	7
Berg & Hayashi 2013	1	1	0	1	0	1	1	0	0	5
Boman et al. 2010	1	1	1	1	1	1	0	0	1	7
Boman & Bodegard 2004	1	1	0	1	1	1	0	0	0	5
Brinkman et al. 2018	1	1	1	1	0	1	1	0	0	6
Burghardt et al. 2019	1	1	1	1	1	1	0	0	0	6
Chan et al. 2020	1	1	1	1	1	1	0	0	1	7
Chaume et al. 2007	1	1	0	1	0	1	0	0	1	5
Clemens et al. 2017	1	1	1	1	0	1	1	1	1	8
Crom et al. 2007	1	1	1	0	1	1	1	1	0	7
De Blank et al. 2016	1	1	1	0	0	1	0	1	1	6
Dieluweit et al. 2011	1	1	1	1	1	1	0	1	0	7
Dowling et al. 2010	1	1	1	0	1	1	0	1	1	7
Dumas et al. 2016	1	1	1	1	1	1	0	1	0	7
Edelstein at al. 2011	1	1	0	1	1	1	1	1	0	7
Effinger et al. 2019	1	1	1	0	1	1	0	1	1	7
Ellenberg et al. 2009	1	1	1	1	1	1	0	1	0	7
Evans et al. 1995	1	1	0	0	1	1	0	0	1	5
Frederiksen et al. 2022	1	1	1	0	1	1	1	1	1	8
Frange et al. 2009	1	1	0	0	0	1	0	0	1	4
Freycon et al. 2013	1	1	0	0	0	1	0	1	1	5
Frobisher et al. 2008	1	1	1	0	1	1	0	1	1	7
Gerhardt et al.2007	1	1	0	1	1	1	0	0	0	5
Gray et al.1992	1	1	0	0	1	1	0	0	0	4
Green et al.1991	1	1	1	1	1	1	1	0	0	7
Guy et al.2018	1	1	1	1	1	1	0	1	1	8
Hayek et al.2020	1	1	1	1	0	1	0	0	1	6
Hays et al.1992	1	1	1	1	1	1	0	0	1	7
Holmqvist et al.2010	1	1	1	1	1	1	0	0	1	7
Howard et al.2014	1	1	0	1	1	1	0	0	1	6
Ishida et al.2014	1	1	1	1	1	1	0	0	0	6
Ishida et al.2011	1	1	1	1	1	1	0	1	1	8
Kieffer et al.2019	1	1	0	1	0	1	0	0	0	4
Kiltie et al.1997	1	1	0	0	0	1	1	0	0	4
Kim et al. 2013	1	0	1	1	1	1	0	0	0	5
Kirchhoff et al.2010	1	1	1	1	1	1	1	0	1	8
Kirchhoff et al. 2011	1	1	1	0	1	1	1	0	1	7
Kirchhoff et al. 2011	1	1	1	0	1	1	1	0	1	7
Korinthenberg et al. 2011	1	1	0	0	1	1	0	0	0	4
Krull et al. 2013	1	1	1	0	1	1	1	0	0	6
Krull et al. 2012	1	1	0	1	1	1	0	0	1	6
Langeveld et al.2003	1	1	1	1	1	1	1	0	1	8
Langeveld et al.2004	1	1	1	1	1	1	0	0	1	7

Lannering et al. 1990	1	1	0	0	0	1	0	0	1	4
Ljungman et al. 2022	1	1	0	1	1	1	1	0	0	6
Lof et al. 2011	1	1	0	1	1	1	1	0	1	7
Lu et al. 2011	1	1	1	0	1	1	0	0	1	6
Marina et al. 2013	1	1	1	1	1	1	0	1	0	7
Maule et al. 2016	1	1	1	0	0	1	0	1	0	5
Meadows et al.1989	1	1	0	1	1	1	1	0	0	6
Mody et al. 2008	1	1	1	0	0	1	1	0	1	6
Mostow et al.1991	1	1	1	1	0	1	0	0	1	6
Mulrooney et al. 2008	1	1	1	1	1	1	1	0	1	8
Nagarajan et al.2003	1	1	1	0	0	1	0	1	1	6
Nathan et al. 2007	1	1	1	0	0	1	0	0	1	5
Nicholson et al. 1992	1	1	1	1	1	1	0	0	0	6
Nicklin et al.2016	1	1	0	1	1	1	1	0	0	6
Nies et al.2017	1	1	0	1	1	1	0	0	0	5

Critical appraisal checklist for studies reporting prevalence data

Q1 - Was the sample frame appropriate to address the target population?

Q2 - Were the study participants sampled in an appropriate way?

Q3 - Was the sample size adequate?

Q4 - Were the study subjects and the setting described in detail?

Q5 - Was the data analysis conducted with sufficient coverage of the identified sample?

Q6 - Were valid methods used for the identification of the condition?

Q7 - Was the condition measured in a standard, reliable way for all participants?

Q8 - Was there appropriate statistical analysis?

Q9 - Was the response rate adequate, and if not, was the low response rate managed appropriately?

Answers: 1 = Yes; 0 = No / Unclear; no answer has been assessed as not applicable (NA).
